# Supplementary material for: A message-passing multi-task architecture for the implicit event and polarity detection
Source: PLoS One. 2021 Mar 1;16(3):e0247704. doi: 10.1371/journal.pone.0247704 (PMC7920338; doi:10.1371/journal.pone.0247704)
Supplement: S1 Dataset — (PDF) [file pone.0247704.s001.pdf]

S1 Dataset

Each sample in the CLIPEval dataset is composed of four parts: a sentence id, a sentence extracted from the English Gigaword corpus, a polarity label and a event label. Some examples in the training set are listed in the following:

1). *1 "And it seems we can walk and run on the mountain for hours again" he added in a recent interview. NEUTRAL OUTDOOR_ACTIVITY*

2). *3 "Every time I have a dispute with my wife, I shout at my sons" he told reporters in Brussels. NEGATIVE COMMUNICATION_ISSUE*

3). *5 "I did it to improve my health and myself" he said. POSITIVE PERSONAL_CARE*

The full dataset are available at `https://alt.qcri.org/semeval2015/task9/`
