# Supplementary material for: A message-passing multi-task architecture for the implicit event and polarity detection
Source: PLoS One. 2021 Mar 1;16(3):e0247704. doi: 10.1371/journal.pone.0247704 (PMC7920338; doi:10.1371/journal.pone.0247704)
Supplement: Attachment — Submitted filename: Response to Reviewers.pdf [file pone.0247704.s002.pdf]

Dear Editor,

The following is my answer to your question for my paper(PONE-D-20-35760)
QA1: Reviewer #1: the present a novel lightweight joint learning paradigm that can pass task-related messages between tasks during training iterations to analyze implicit sentiment. The overall application is supported with convincing quantitative experimental results and comparisons on available datasets. The author should to explain weather the Baseline models is the state-of- the -art. At same time, there need improvements (English usage and grammar check is necessary)

Response:
Thank you for your comments.
Three baselines are state-of-the-art models.
We clarified this in the section of "Baseline models" and add corresponding references after each model name.
We proof read the paper carefully and corrected grammar and spelling errors.

Yours sincerely ,
Chunli
